# Supplementary material for: FRET-FISH probes chromatin compaction at individual genomic loci in single cells
Source: Nat Commun. 2022 Nov 5;13:6680. doi: 10.1038/s41467-022-34183-y (PMC9637210; doi:10.1038/s41467-022-34183-y)
Supplement: Supplementary file 1 — Supplementary Information [file 41467_2022_34183_MOESM1_ESM.pdf]

SUPPLEMENTARY INFORMATION

FRET-FISH probes chromatin compaction at individual genomic loci in single cells

Ana Mota, Szymon Berezicki, Erik Wernersson, Luuk Harbers, Xiaoze Li-Wang, Katarina Gradin, Christiane Peuckert, Nicola Crosetto, Magda Bienko

1. Supplementary Figures pg. 2
2. Supplementary Tables pg. 25
3. Supplementary References pg. 26

1. Supplementary Figures

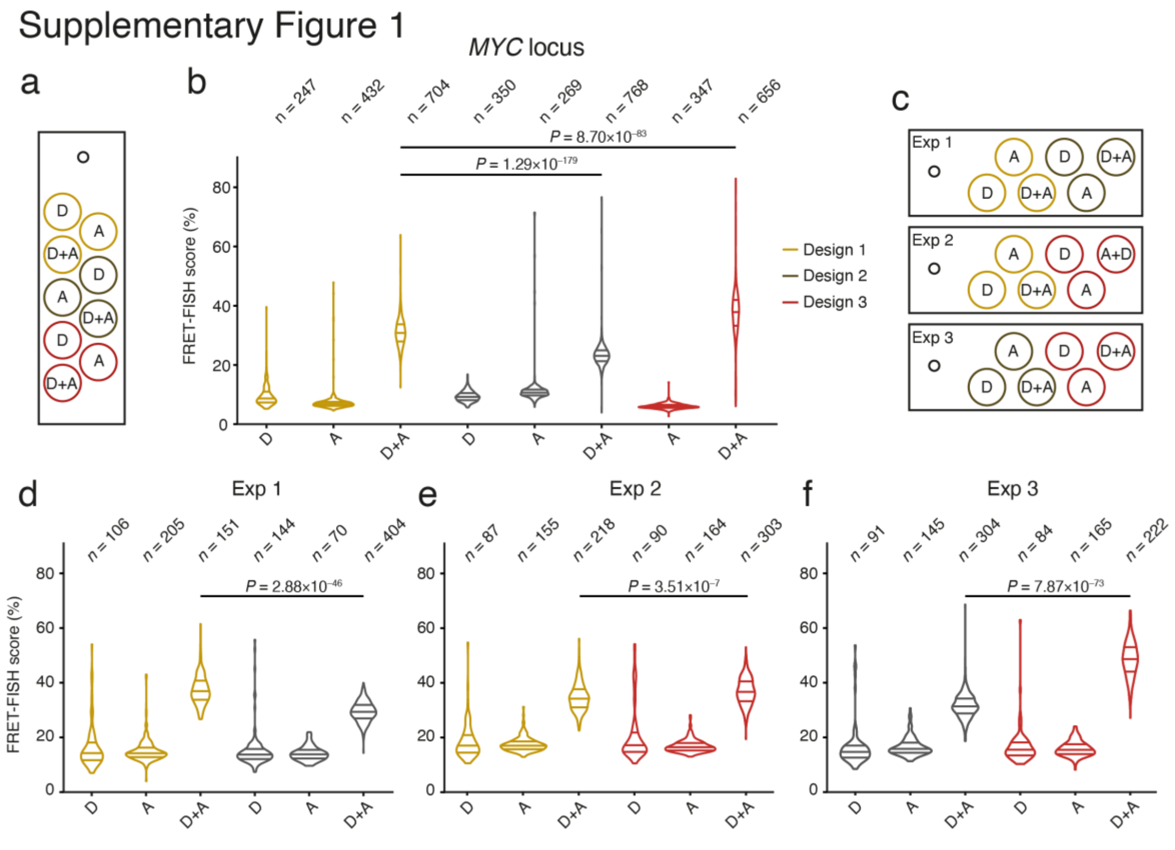

Supplementary Fig. 1. FRET-FISH probe design optimization. (**a**) Scheme of the custom-designed chambered coverslip system used to compare the three FRET-FISH probe designs shown in **Fig. 1b**. D, wells containing cells hybridized with FRET-FISH probes containing only FRET donor (D) oligos. A, wells containing cells hybridized with FRET-FISH probes containing only FRET acceptor (A) oligos. D+A, wells containing cells hybridized with FRET-FISH probes containing both D and A oligos. (**b**) Distributions of the FRET-FISH scores (Cy3 excitation, Cy5 emission) obtained with a FRET-FISH probe targeting the *MYC* gene locus in human HAP1 cells, for each of the three probe designs shown in **Fig. 1b**. D, probes containing only donor oligos. A, probes containing only acceptor oligos. D+A, probes containing both donor and acceptor oligos. *n*, number of FRET signals analyzed. *P*, Wilcoxon test, two-tailed. (**c**) Same as in (a), but for different custom-made 6-chamber coverslips. Exp, experiment. (**d**) Same as in (b), but for the experimental setup Exp1 shown in (c). (**e**) Same as in (b), but for the experimental setup Exp2 shown in (c). (**f**) Same as in (b), but for the experimental setup Exp3 shown in (c). In (b) and (d-f), violins extend from minimum to maximum and horizontal lines represent (from top to bottom) the 75th percentile, the median, and the 25th percentile of each

distribution. Source data for all the plots shown in the figure are provided as a separate Source Data file.

Supplementary Figure 2

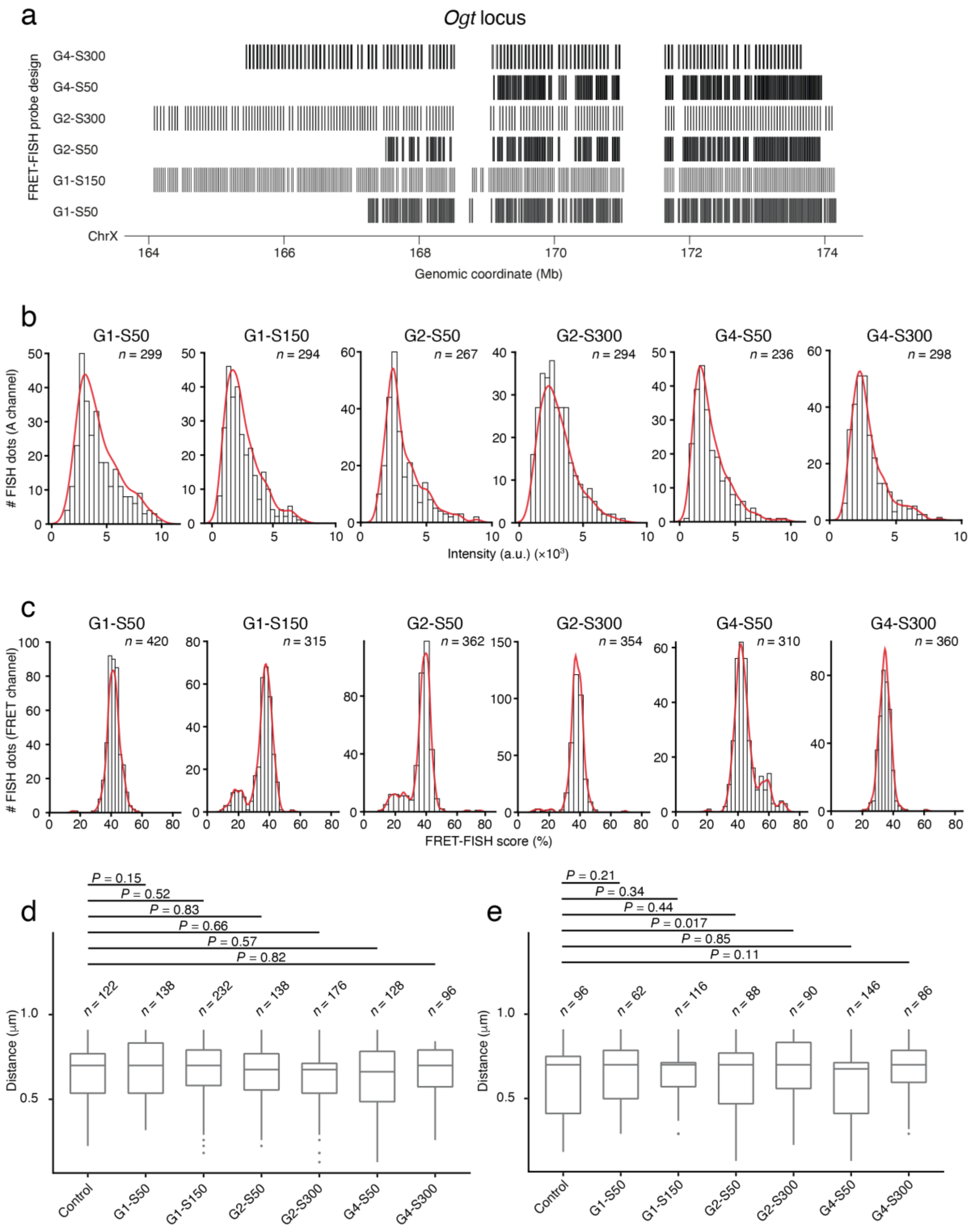

Supplementary Fig. 2. FRET-FISH probe design optimization. (**a**) Schematic representation of the genomic distribution of A and D oligos for different FRET-FISH probe designs targeting the mouse *Ogt* locus on chrX. Each vertical black bar corresponds to one oligo in the probe (for simplicity, A and D oligos are not distinguished). For a schematic description of the different

probe designs, see **Fig. 1e-j**. (**b**) Distributions of the number of FISH dots detected in the A channel (Cy3) obtained for the different *Ogt* probe designs described in **Fig. 1e-j**, in female mouse embryonic fibroblasts (MEFs). Red lines, kernel density estimation function. *n*, number of nuclei analyzed. (**c**) Same as in (b) but for FISH dots detected in the FRET channel (Cy3 excitation, Cy5 emission) in mouse NIH3T3 cells. (**d**) Distributions of physical (3D) distances measured between two probes flanking the genomic region targeted by different *Ogt* probe designs in female MEFs (see **Supplementary Data 1** for the list of sequences and genomic coordinates of the oligos in the flanking probes). Only A oligos in the Ogt probes were co-hybridized with the two flanking probes. Boxplots extend from the 25th to the 75th percentile, horizontal bars represent the median, and whiskers extend from –1.5×IQR to +1.5×IQR from the closest quartile, where IQR is the inter-quartile range. Gray dots, outliers. In each boxplot, the minimum and maximum are defined, respectively, by the uppermost and lowermost outlier dot or extremity of the corresponding whisker. *n*, number of FRET signals analyzed. *P*, Wilcoxon test, two-tailed. (**e**) Same as in (d) but with both A and D oligos in the Ogt probes co-hybridized with the flanking probes. In (b-e), *n* represents the number of FRET signals analyzed. Source data for all the plots shown in the figure are provided as a separate Source Data file.

Supplementary Figure 3

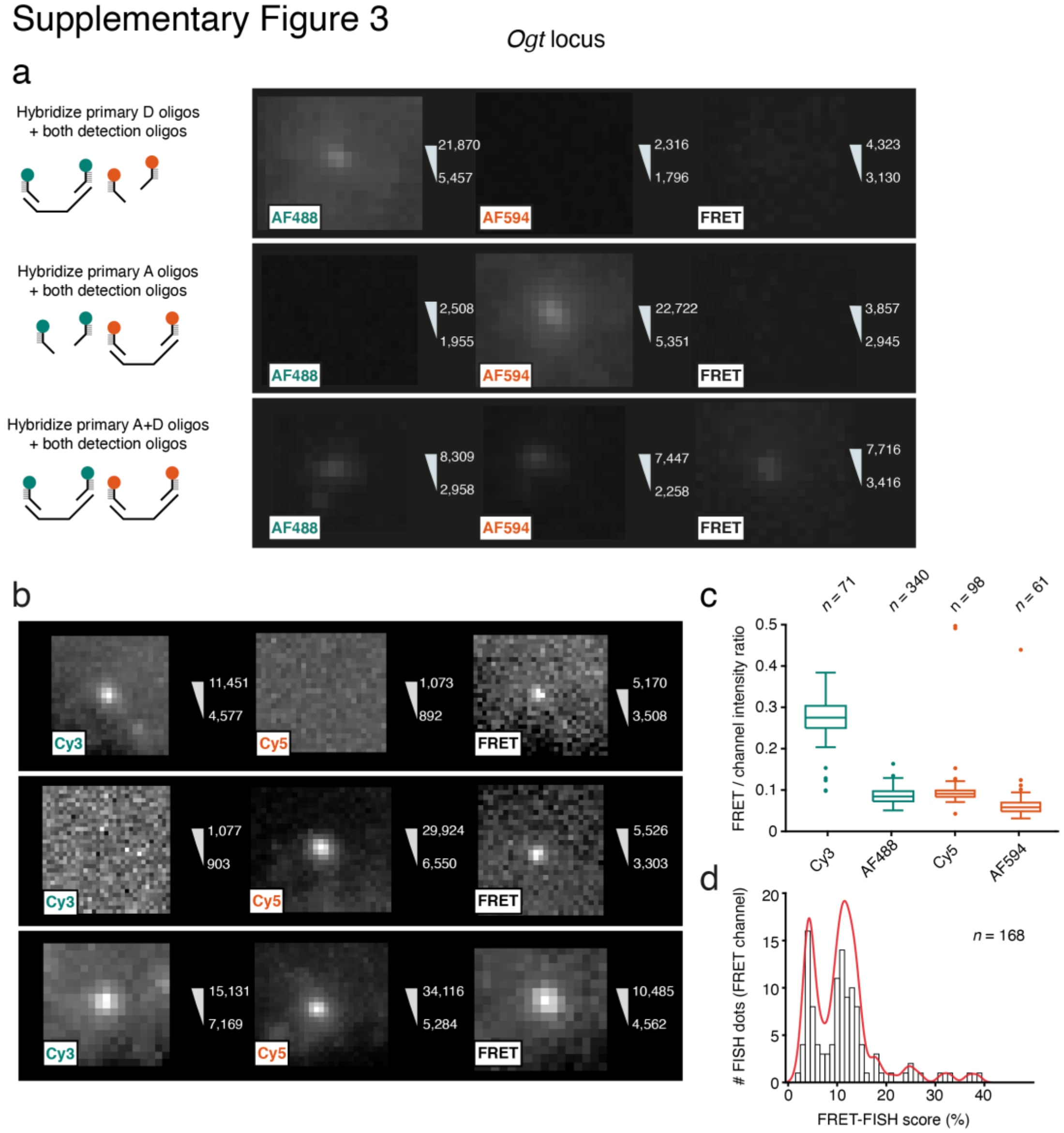

Supplementary Fig. 3. Comparison between different donor-acceptor dye pairs. (**a**) Example of a signal obtained with the G1-S150 *Ogt* probe shown in **Fig. 1e** by hybridizing either only the primary D oligos (top) or only the primary A oligos (middle) or both (bottom), together with both detection oligos, in female mouse embryonic fibroblasts (MEFs). Alexa Fluor 488 (AF488) and Alexa Fluor 594 (AF594) were used as FRET donor and acceptor dyes, respectively. The white triangles and numbers near each panel represent the minimum (bottom) and maximum (top) intensity values in the corresponding image on the left. (**b**) Same as in (a) but using Cy3 and Cy5 as FRET donor and acceptor dyes, respectively. (**c**) Distributions of the ratio between the intensity measured in the FRET channel and the intensity measured in the indicated channel, for the same G1-S150 *Ogt* probe shown in (a) hybridized in female MEFs. Boxplots extend from the 25th to the 75th percentile, horizontal bars represent the median, and

whiskers extend from –1.5×IQR to +1.5×IQR from the closest quartile, where IQR is the inter-quartile range. Green and orange dots, outliers. In each boxplot, the minimum and maximum are defined, respectively, by the uppermost and lowermost outlier dot or extremity of the corresponding whisker. *n*, number of FRET signals analyzed. (**d**) Distributions of the number of FISH dots detected in the FRET channel (AF488 excitation, AF594 emission) for the same G1-S150 *Ogt* probe shown in (a) in female MEFs. Red line, kernel density estimation function. *n*, number of FRET signals analyzed. Source data for all the plots shown in the figure are provided as a separate Source Data file.

Supplementary Figure 4

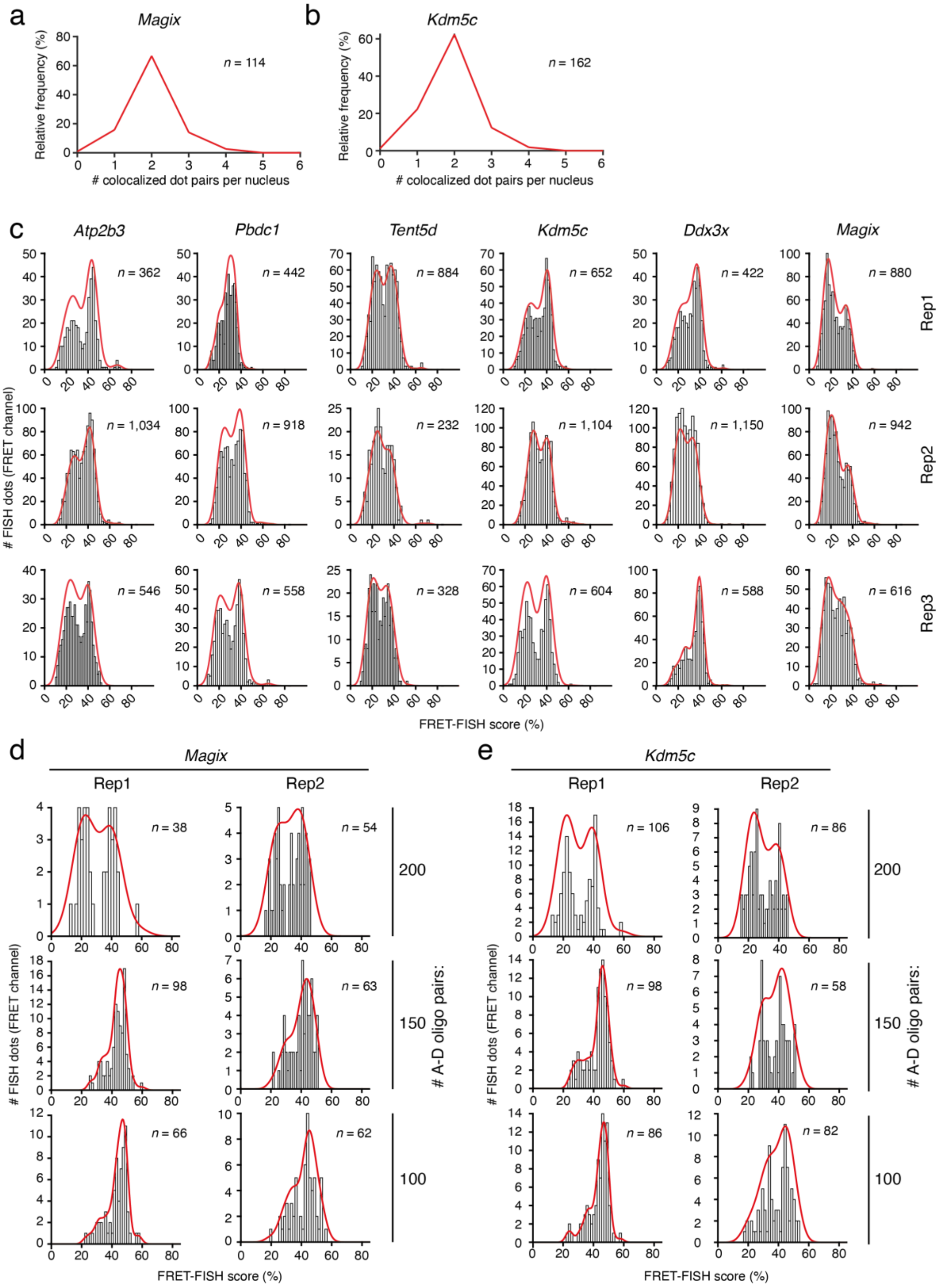

Supplementary Fig. 4. FRET-FISH reproducibility and sensitivity. (**a**) Distribution of the number of co-localized acceptor and donor FISH dot pairs per nucleus, in female mouse

embryonic fibroblasts (MEFs) hybridized with the FRET-FISH probe targeting the *Magix* gene on chrX. *n*, number of nuclei analyzed. (**b**) Same as in (a) but for the FRET-FISH probe targeting the *Kdm5c* locus on chrX. (**c**) Distributions of the number of FISH dots detected in the FRET channel (AF488 excitation, AF594 emission) using FRET-FISH probes targeting the indicated genes on mouse chrX, in female MEFs in three replicate (Rep) experiments. *n*, number of FRET signals analyzed. Red lines, kernel density estimation function. (**d**) Distributions of the number of FISH dots detected in the FRET channel (AF488 excitation, AF594 emission) for three FRET-FISH probes containing decreasing numbers of D-A oligo pairs, targeting the *Magix* locus in female MEFs. Red lines, kernel density estimation function. (**e**) Same as in (d) but for three probes targeting the *Kdm5c* locus. See **Supplementary Data 1** for the list of oligos in each probe. Source data for all the plots shown in the figure are provided as a separate Source Data file.

Supplementary Figure 5

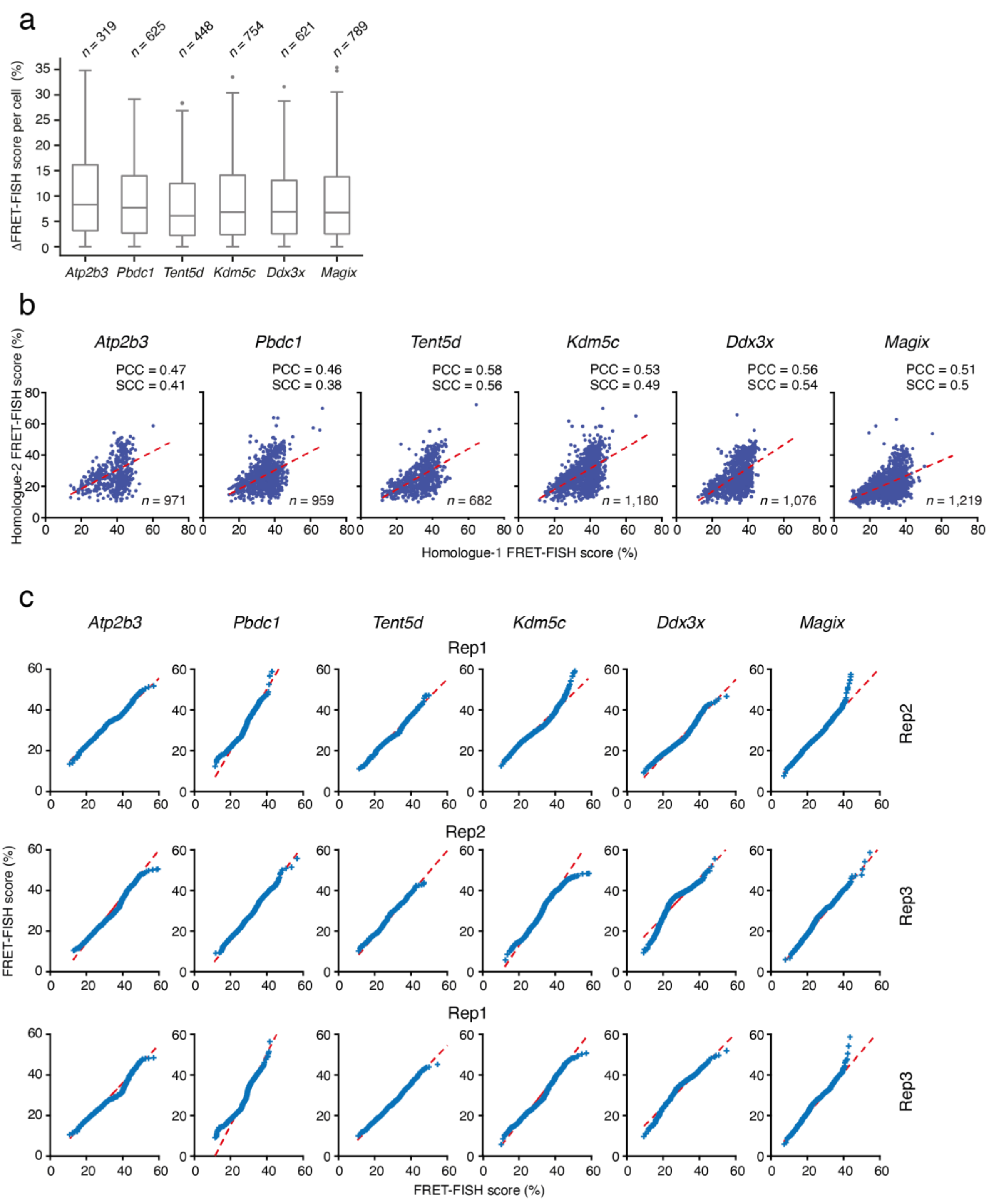

Supplementary Fig. 5. FRET-FISH distinguishes between homologue loci in the same cell and is highly reproducible. (**a**) Distributions of the absolute difference (delta) in the FRET-FISH score between the two homologue loci in the same cell, for each of the six FRET-FISH probes targeting the indicated genes on chrX in mouse female fibroblasts (MEFs) (see **Supplementary Data 1** for the list of oligos in each probe). Boxplots extend from the 25th to

the 75th percentile, horizontal bars represent the median, and whiskers extend from –1.5×IQR to +1.5×IQR from the closest quartile, where IQR is the inter-quartile range. Grey dots, outliers. In each boxplot, the minimum and maximum are defined, respectively, by the uppermost and lowermost outlier dot or extremity of the corresponding whisker. *n*, number of FRET signals analyzed. (**b**) Scatterplots of the FRET-FISH score measured for one of the two homologues *versus* the other in the same nucleus, for the same six genes shown in (a), in female MEFs. PCC, Pearson's correlation coefficient. SCC, Spearman's correlation coefficient. Dashed red lines, linear regression fit. *n*, number of homologue pairs analyzed. (**c**) Quantile-quantile plots of the FRET-FISH scores obtained in three independent replicate (Rep) experiments in female MEFs, for the same six genes shown in (a). Dashed red lines, linear regression fit. Source data for all the plots shown in the figure are provided as a separate Source Data file.

Supplementary Figure 6

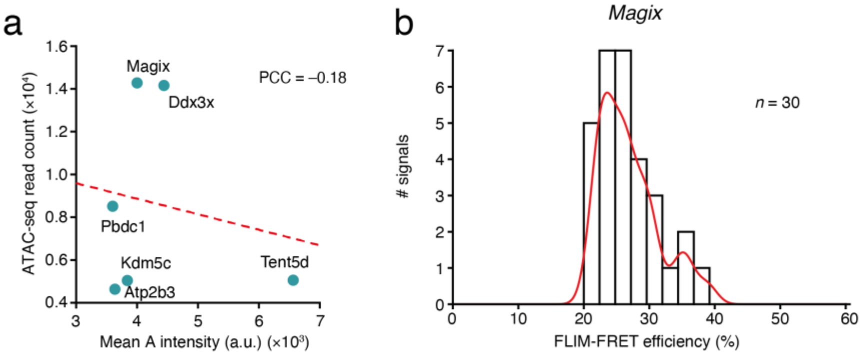

Supplementary Fig. 6. Comparison between FRET-FISH and ATAC-seq (**a**) Correlation between the mean intensity measured in FRET-FISH acceptor channel (AF594 excitation and emission) and the ATAC-seq read counts in the corresponding genomic regions, for the six loci probed by FRET-FISH on chrX in female mouse embryonic fibroblasts (MEFs). PCC, Pearson's correlation coefficient. *P*, Wilcoxon test, two-tailed. Dashed red line, linear regression fit. (**b**) Distribution of FRET efficiencies calculated from fluorescence lifetime imaging microscopy (FLIM) data (see **Methods**), for the *Magix* locus on chrX imaged in female MEFs. Red lines, kernel density estimation function. *n*, number of FRET signals analyzed. Source data for all the plots shown in the figure are provided as a separate Source Data file.

Supplementary Figure 7

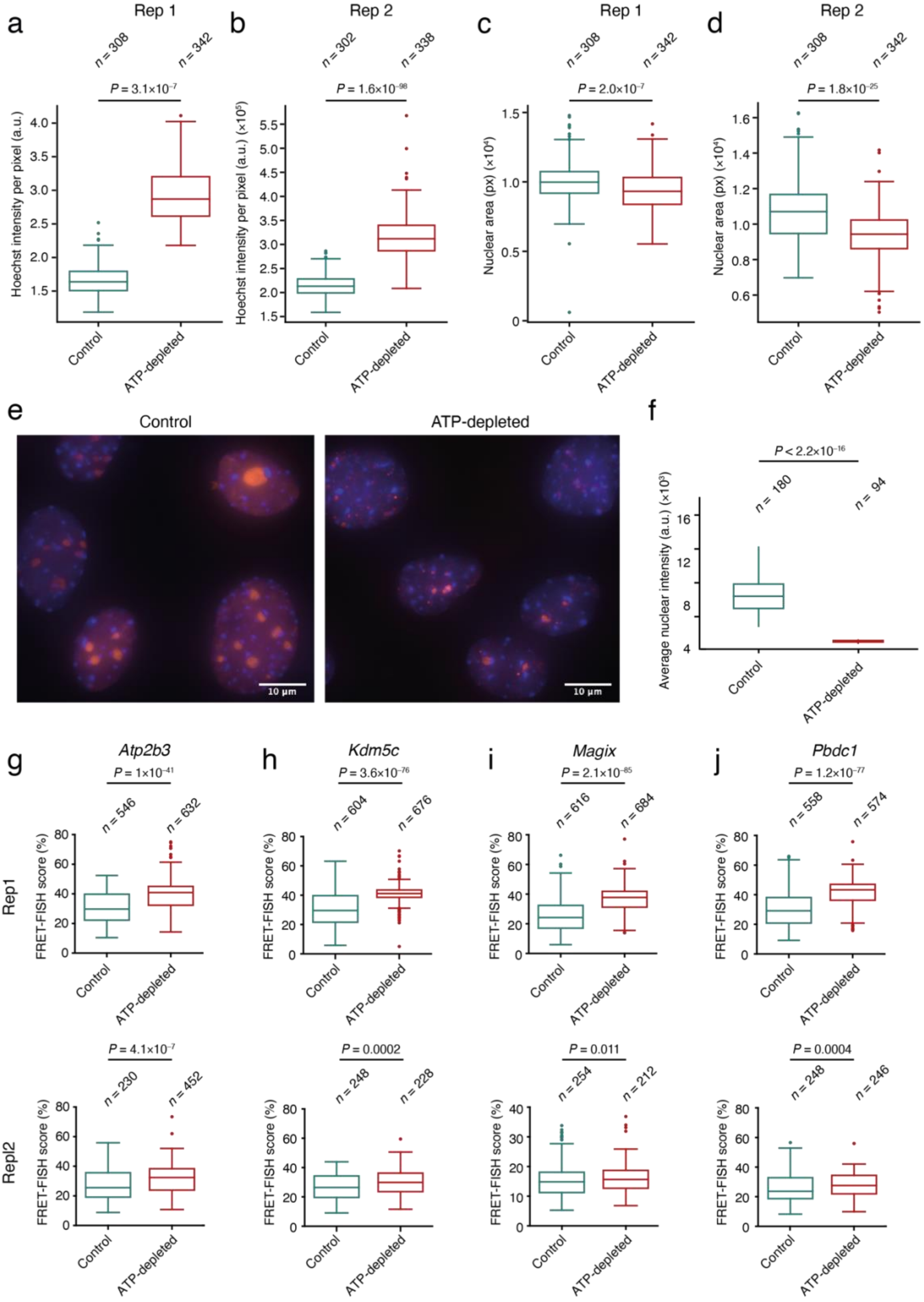

Supplementary Fig. 7. FRET-FISH detects local chromatin compaction changes induced by ATP depletion. (**a, b**) Distribution of the nuclear fluorescence intensity of the DNA intercalator

dye, Hoechst 33342, in two replicate (Rep) experiments in which female mouse embryonic fibroblasts (MEFs) were treated (ATP depletion) or not (Control) with a combination of sodium azide and 2-deoxy-d-glucose, which causes ATP depletion and, consequently, global chromatin condensation. *n*, number of FRET signals analyzed. *P*, Wilcoxon test, two-tailed. (**c, d**) Same as in (a, b) but showing the distributions of the area of segmented nuclei (2D projections). (**e**) Nascent RNA (orange) visualized in the one of the replicate experiments analyzed in (a-c). Blue, DNA stained with Hoechst 33342. (**f**) Quantification of nascent transcripts (average nuclear fluorescent intensity in the red channel) in the microscopy images exemplified in (e). *n*, number of nuclei analyzed. *P*, Wilcoxon test, two-tailed. (**g-j**) Distributions of the FRET-FISH scores obtained in two replicate (Rep) ATP depletion experiments, for four of the six genes on chrX studied with FRET-FISH. *n*, number of FRET signals analyzed. *P*, Wilcoxon test, two-tailed. In (a-d) and (f-j), boxplots extend from the 25th to the 75th percentile, horizontal bars represent the median, and whiskers extend from –1.5×IQR to +1.5×IQR from the closest quartile, where IQR is the inter-quartile range. Turquois and red dots, outliers. In each boxplot, the minimum and maximum are defined, respectively, by the uppermost and lowermost outlier dot or extremity of the corresponding whisker. Source data for all the plots shown in the figure are provided as a separate Source Data file.

Supplementary Figure 8

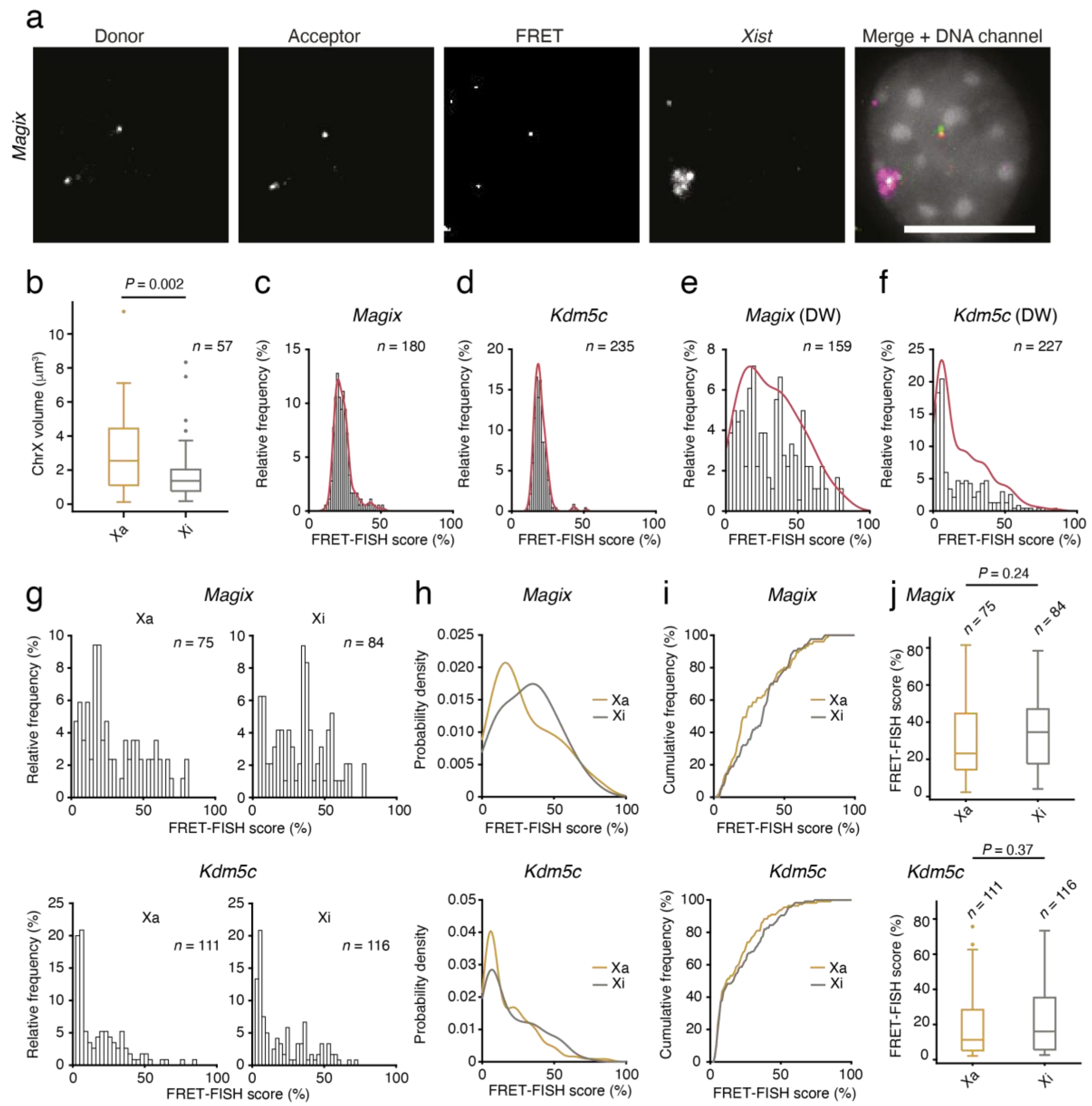

Supplementary Fig. 8. FRET-FISH detects no major difference in local compaction between the active and inactive chrX copy in female cells. (**a**) Maximum z-projections exemplifying the results of combined FRET-FISH with a probe targeting the *Magix* locus on chrX and single-molecule FISH (smFISH) with a probe targeting *Xist* RNA, which marks the inactive chrX copy. Gray, DNA stained with Hoechst 33342. Scale bar, 10 μm. (**b**) Distribution of active (Xa) and inactive (Xi) chrX territory in female MEFs cells. The territories were visualized with our previously described ‘chromosome spotting’ approach[1] using probes targeting multiple evenly separated loci on chrX. *n*, number of nuclei analyzed. *P*, Wilcoxon test, two-tailed. (**c**) FRET-FISH score distribution for female MEFs co-hybridized with a FRET-FISH probe targeting the

Magix gene on chrX and an smFISH targeting *Xist*. *n*, number of FRET signals analyzed. Red lines, kernel density estimation function. (**d**) Same as in (c) but for the *Kdm5c* gene on chrX. (**e, f**) Same as in (c) and (d), respectively, but after deconvolving the corresponding images with our deconvolution software Deconwolf (DW)[2]. (**g**) FRET-FISH score distributions for the *Magix* (top) and *Kdm5c* (bottom) loci on the active (Xa) and inactive (Xi) chrX copy in female MEFs. *n*, number of FRET signals analyzed. (**h**) Kernel density estimation functions for the corresponding histograms in (g). *n*, number of FRET signals analyzed. (**i**) Cumulative frequency curves for the corresponding histograms in (g). (**j**) Same as in (g) but shown as boxplots. *n*, number of FRET signals analyzed. *P*, Wilcoxon test, two-tailed. In (b) and (j), boxplots extend from the 25th to the 75th percentile, horizontal bars represent the median, and whiskers extend from –1.5×IQR to +1.5×IQR from the closest quartile, where IQR is the inter-quartile range. Yellow and gray dots, outliers. In each boxplot, the minimum and maximum are defined, respectively, by the uppermost and lowermost outlier dot or extremity of the corresponding whisker. Source data for all the plots shown in the figure are provided as a separate Source Data file.

Supplementary Figure 9

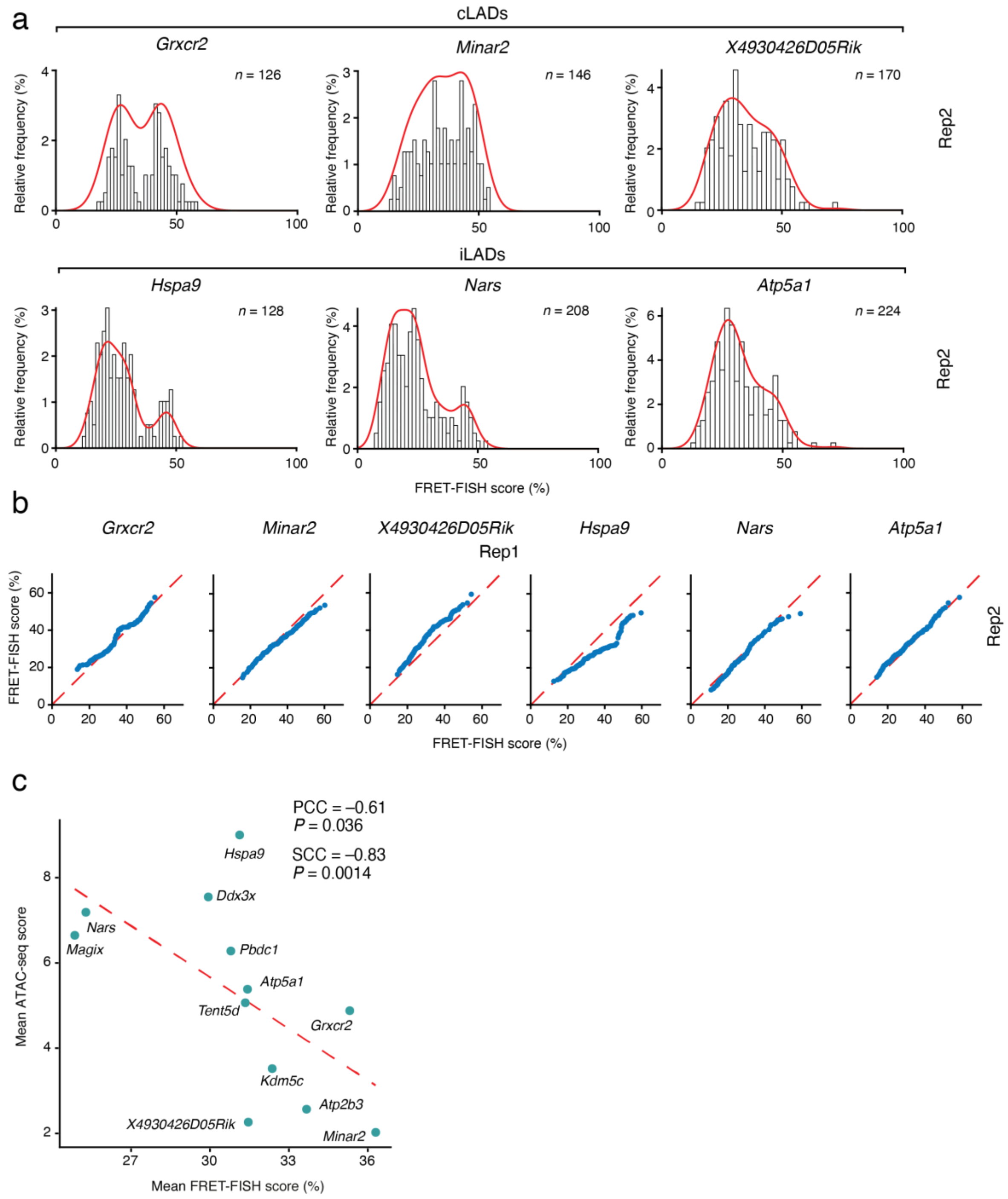

Supplementary Fig. 9. FRET-FISH reproducibly detects local chromatin compaction at different loci on chr18. (**a**) FRET-FISH score distributions for six genes on chr18 probed with FRET-FISH in one of two replicate (Rep) experiments in female mouse embryonic fibroblasts (MEFs). *n*, number of FRET signals analyzed. Red lines, kernel density estimation function. (**b**) Quantile-quantile plots showing the reproducibility of the FRET-FISH score distributions shown in (a). Dashed red lines, linear regression fit. (**c**) Correlation between the mean FRET-

FISH score and the mean ATAC-seq score of the corresponding genomic regions, for all the loci probed by FRET-FISH on chr18 and chrX in female MEFs. PCC, Pearson's correlation. SCC, Spearman's correlation coefficient. *P*, Wilcoxon test, two-tailed. Dashed red line, linear regression fit. Source data for all the plots shown in the figure are provided as a separate Source Data file.

Supplementary Figure 10

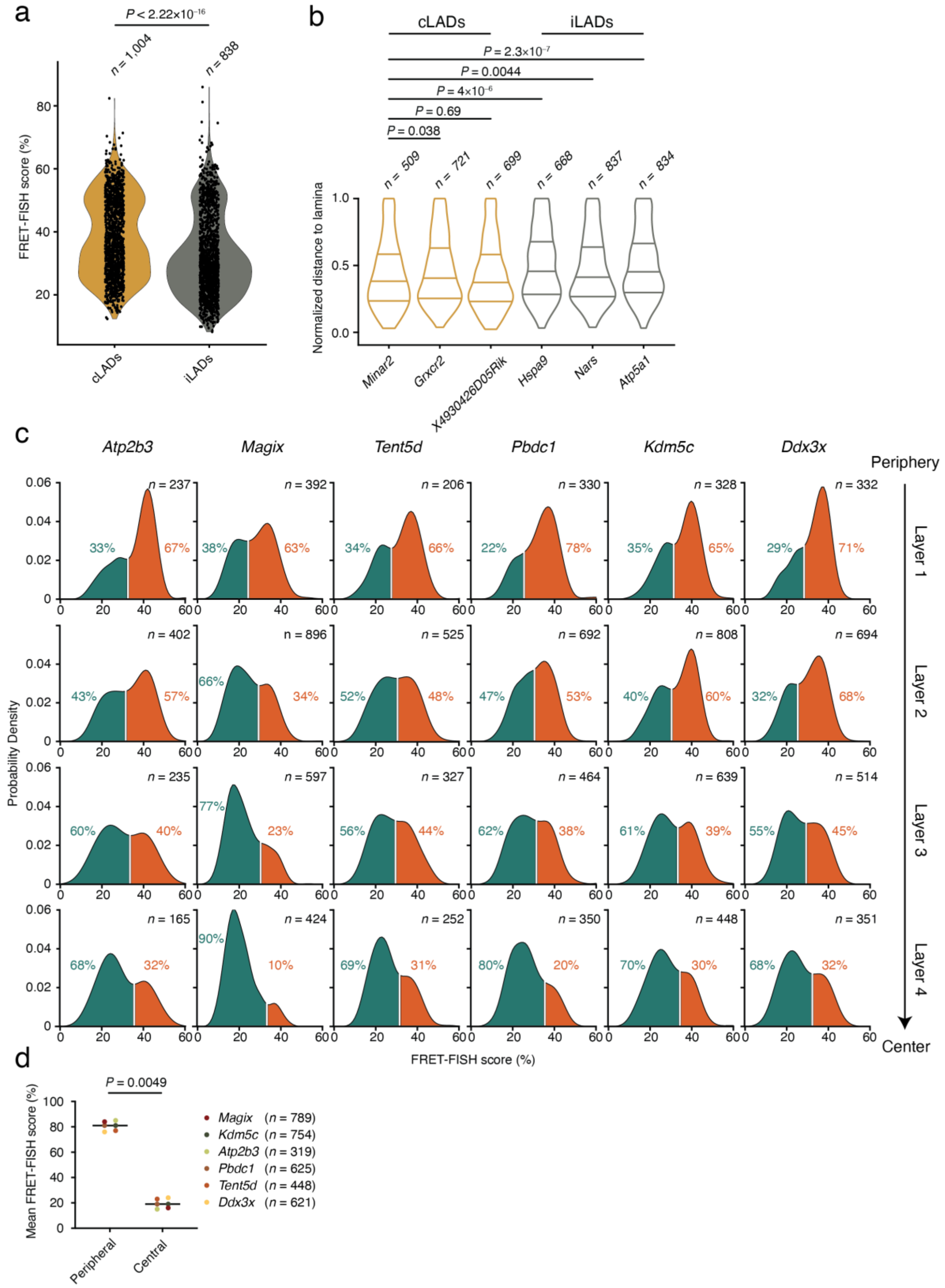

Supplementary Fig. 10. Local chromatin compaction depends on the radial distance of a locus from the nuclear lamina. (**a**) FRET-FISH score distributions for the three genes on chr18 located

within constitutive lamina-associated domains (cLADs) and the three genes on chr18 located in inter-LAD regions (iLADs) shown in (b), assessed by FRET-FISH in female mouse embryonic fibroblasts (MEFs). (**b**) Distributions of the normalized distance to lamina for three loci on chr18 located in cLADs and three loci on chr18 located in iLADs. In (a) and (b), violins extend from the minimum to the maximum value. Black dots in (a) represent individual FRET-FISH score measurements. *n*, number of FRET signals analyzed. *P*, Wilcoxon test, two-tailed. (**c**) FRET-FISH score distributions calculated separately for four concentric nuclear layers of equal size (see **Methods**), using the same dataset on which the plots in (a) and (b) are based. (**d**) Mean FRET-FISH score for the peripheral and central homologue in each cell analyzed, for the six genes on chrX shown in (c). Each dot represents the average FRET-FISH score for that gene/homologue. Horizontal black lines represent the mean. *n*, number of FRET signals analyzed. *P*, Wilcoxon test, two-tailed. Source data for all the plots shown in the figure are provided as a separate Source Data file.

Supplementary Figure 11

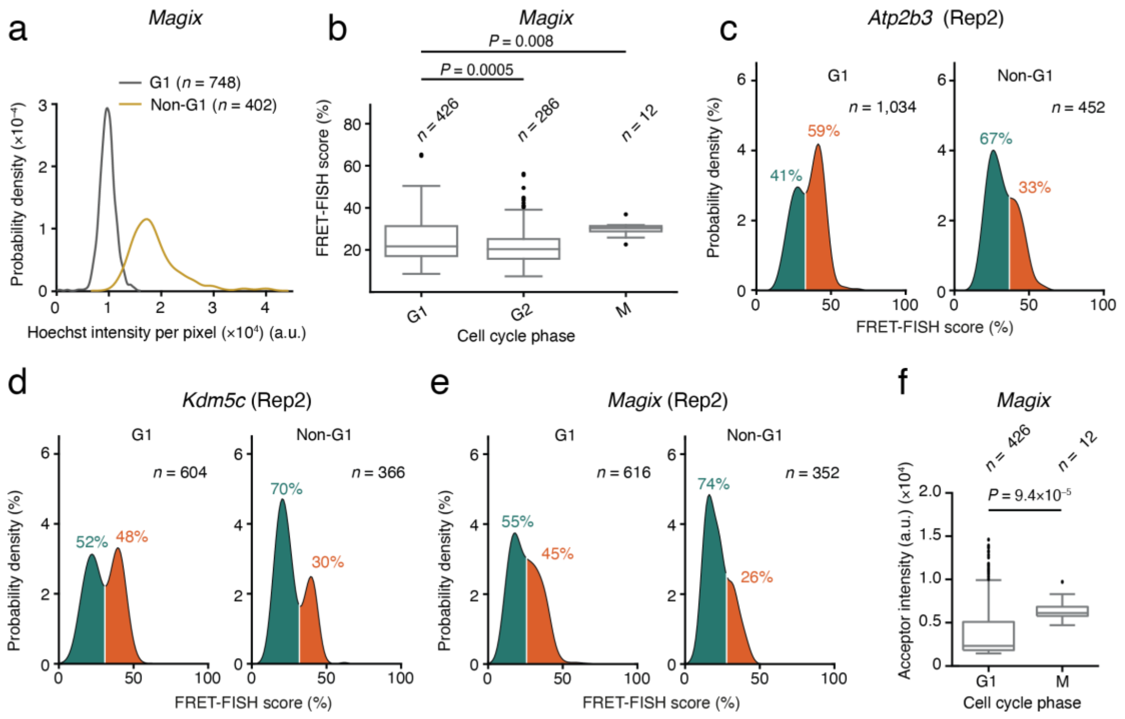

Supplementary Fig. 11. FRET-FISH detects local chromatin compaction changes during the cell division cycle. (**a**) Distribution of the fluorescence intensity of the DNA intercalator dye, Hoechst 33342, in the nucleus of female mouse embryonic fibroblasts (MEFs) hybridized with the FRET-FISH probe targeting the *Magix* locus on chrX and classified as belonging to the G1 or another (non-G1) phase of the cell cycle (see **Methods** for how cells were classified as G1 or non-G1). *n*, number of nuclei analyzed. *P*, Wilcoxon test, two-tailed. (**b**) FRET-FISH score distribution for the *Magix* locus in female MEFs in different phases of the cell cycle. Mitotic cells (M) were identified by visually inspecting all the fields of view, whereas G1 and G2 cells were automatically identified based on their DNA content (see (a)). *n*, number of FRET signals analyzed. *P*, Wilcoxon test, two-tailed. (**c-e**) FRET-FISH score distributions for three loci on chrX assessed by FRET-FISH in female MEFs, for cells classified as G1 or non-G1 based on their DNA content (see (a)), in one of two replicate (Rep) experiments. The inflection point in the bimodal distribution was used to automatically distinguish between homologue loci with more compact (red) or less compact (green) chromatin. The percentages on top of each peak indicate the proportion of all the FRET signals assigned to that group. *n*, number of FRET signals analyzed. (**f**) Distribution of the *Magix* FRET-FISH probe acceptor intensity (AF594 excitation and emission) in female MEFs in G1 or M phase. *n*, number of FRET signals analyzed. *P*, Wilcoxon test, two-tailed. In (b) and (f), boxplots extend from the 25th to the 75th

percentile, horizontal bars represent the median, and whiskers extend from –1.5×IQR to +1.5×IQR from the closest quartile, where IQR is the inter-quartile range. Black and gray dots, outliers. In each boxplot, the minimum and maximum are defined, respectively, by the uppermost and lowermost outlier dot or extremity of the corresponding whisker. Source data for all the plots shown in the figure are provided as a separate Source Data file.

Supplementary Figure 12

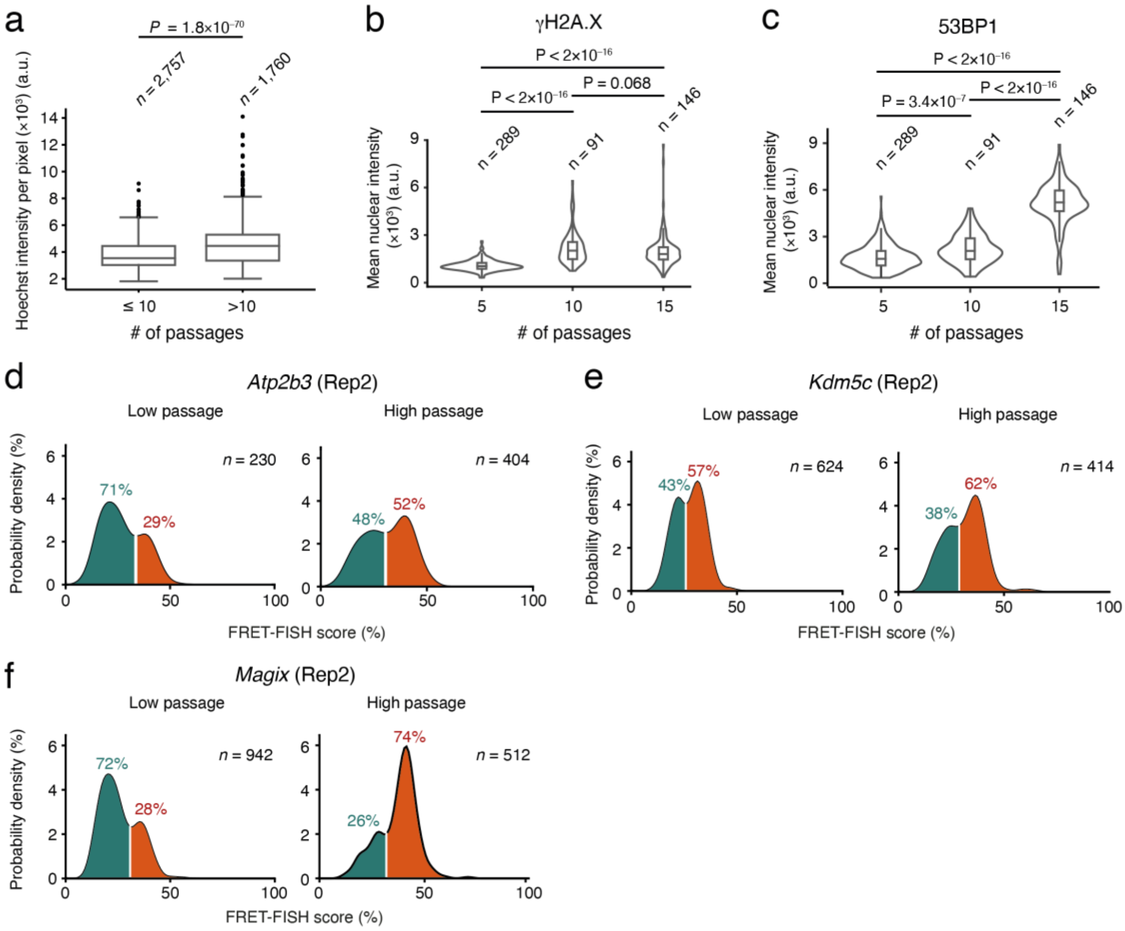

Supplementary Fig. 12. FRET-FISH detects local chromatin compaction changes during prolonged cell culturing *in vitro*. (**a**) Distribution of the nuclear fluorescence intensity of the DNA intercalator dye, Hoechst 33342, in female mouse embryonic fibroblasts (MEFs) cultured for less or more than 10 passages. Boxplots extend from the 25th to the 75th percentile, horizontal bars represent the median, and whiskers extend from –1.5×IQR to +1.5×IQR from the closest quartile, where IQR is the inter-quartile range. Black dots, outliers. In each boxplot, the minimum and maximum are defined, respectively, by the uppermost and lowermost outlier dot or extremity of the corresponding whisker. *n*, number of nuclei analyzed. *P*, Wilcoxon test, two-tailed. (**b**) Distributions of nuclear fluorescence intensity at increasing in vitro passage numbers, for MEFs subjected to immunofluorescence with an antibody against phosphorylated histone H2A.X (γH2A.X). Violins extend from minimum to maximum, boxplots extend from the 25th to the 75th percentile, horizontal bars represent the median, and whiskers extend from –1.5×IQR to +1.5×IQR from the closest quartile, where IQR is the inter-quartile range. *n*, number of nuclei analyzed. *P*, Wilcoxon test, two-tailed. (**c**) Same as in (b) but for the DNA

damage marker 53BP1. (**d-f**) FRET-FISH score distributions for three loci on chrX in female MEFs cultured for less (Low passage) or more (High passage) than 10 passages, in one of two replicate (Rep) experiments. The inflection point in each bimodal distribution was used to separate between the lower (green) and higher (orange) FRET-FISH score mode corresponding, respectively, to a less and more compact chromatin state. The percentages indicate the proportion of FRET signals in each group. *n*, number of FRET signals analyzed. Source data for all the plots shown in the figure are provided as a separate Source Data file.

2. Supplementary Tables

Supplementary Table 1. Probe span and number of D and A oligos in each of the probes used in this study. For a visual description of each probe design, see **Fig. 1e-j**.

Gene name	Probe design	Probe span (bp)	# D oligos	# A oligos
Atp2b3	G1-S150	104,848	225	225
Atp5a1	G1-S150	100,842	417	417
Ddx3x	G1-S150	128,607	221	221
Grxcr2	G1-S150	104,145	206	206
Hspa9	G1-S150	94,742	207	207
Kdm5c	G1-S150	108,940	233	233
Ogt	G1-S50	69,213	221	220
Ogt	G1-S150	100,693	202	201
Ogt	G2-S50	64,326	220	220
Ogt	G2-S300	100,330	190	190
Ogt	G4-S50	48,600	220	219
Ogt	G4-S300	82,206	224	223
Magix	G1-S150	104,359	237	236
Minar2	G1-S150	100,619	208	208
Nars	G1-S150	94,318	212	212
Pbdc1	G1-S150	108,992	209	209
4930426D05Rik	G1-S150	93,995	209	209

Supplementary Table 2. List of filters and dichroic mirrors for each dye used in FRET-FISH.

Dye	Excitation filter	Dichroic mirror	Emission filter
Hoechst 33342	390/22 (Lumencor)	440/40 (custom-made Polychroic 1 from Chroma)	FF01-447/60-25 (Semrock)
AF488	FF01-494/20-25 (Semrock)	FF506-Di 03-25x36 (Semrock)	FF01-527/20-25 (Semrock)
Cy3	FF01-534/20-25 (Semrock)	570/20 (custom-made Polychroic 1 from Chroma)	FF01-567/15-25 (Semrock)
AF594	FF01-586/20-25x5 (Semrock)	630/27 (custom-made Polychroic 2 from Chroma)	FF01-628/32-25 (Semrock)
Cy5	631/28 (Lumencor)	675/29 (custom-made Polychroic 1 from Chroma)	FF01-676/29-25 (Semrock)

3. Supplementary References

1. Gelali, E. *et al.* iFISH is a publically available resource enabling versatile DNA FISH to study genome architecture. *Nat. Commun.* **10**, 1636 (2019).
2. Wernersson, E. *et al.* Deconwolf enables high-performance deconvolution of widefield fluorescence microscopy images. at https://doi.org/10.21203/rs.3.rs-1303463/v1 (2022).
