# Supplementary material for: FRET-FISH probes chromatin compaction at individual genomic loci in single cells
Source: Nat Commun. 2022 Nov 5;13:6680. doi: 10.1038/s41467-022-34183-y (PMC9637210; doi:10.1038/s41467-022-34183-y)
Supplement: Supplementary file 3 — Description of Additional Supplementary Files [file 41467_2022_34183_MOESM3_ESM.pdf]

Description of Additional Supplementary Files

Supplementary Data 1. List of oligos composing the FRET-FISH probes used in this study. Because of its size, this table is provided as a separate Excel file.

Supplementary Data 2. Comparison of denaturation conditions in various oligo-based DNA FISH protocols. Because of its size, this table is provided as a separate Excel file.

Supplementary Data 3. List of datasets generated in this study. Because of its size, this table is provided as a separate Excel file.
