# Supplementary material for: FRET-FISH probes chromatin compaction at individual genomic loci in single cells
Source: Nat Commun. 2022 Nov 5;13:6680. doi: 10.1038/s41467-022-34183-y (PMC9637210; doi:10.1038/s41467-022-34183-y)
Supplement: Supplementary file 7 — Reporting Summary [file 41467_2022_34183_MOESM7_ESM.pdf]

nature research

Corresponding author(s): Magda Bienko
Last updated by author(s): Sep 28, 2022

Reporting Summary

Nature Research wishes to improve the reproducibility of the work that we publish. This form provides structure for consistency and transparency in reporting. For further information on Nature Research policies, see our Editorial Policies and the Editorial Policy Checklist.

Statistics

For all statistical analyses, confirm that the following items are present in the figure legend, table legend, main text, or Methods section.

n/a	Confirmed	
☐	☒	The exact sample size (*n*) for each experimental group/condition, given as a discrete number and unit of measurement
☐	☒	A statement on whether measurements were taken from distinct samples or whether the same sample was measured repeatedly
☐	☒	The statistical test(s) used AND whether they are one- or two-sided<br>*Only common tests should be described solely by name; describe more complex techniques in the Methods section.*
☒	☐	A description of all covariates tested
☒	☐	A description of any assumptions or corrections, such as tests of normality and adjustment for multiple comparisons
☐	☒	A full description of the statistical parameters including central tendency (e.g. means) or other basic estimates (e.g. regression coefficient) AND variation (e.g. standard deviation) or associated estimates of uncertainty (e.g. confidence intervals)
☐	☒	For null hypothesis testing, the test statistic (e.g. *F*, *t*, *r*) with confidence intervals, effect sizes, degrees of freedom and *P* value noted<br>*Give P values as exact values whenever suitable.*
☒	☐	For Bayesian analysis, information on the choice of priors and Markov chain Monte Carlo settings
☒	☐	For hierarchical and complex designs, identification of the appropriate level for tests and full reporting of outcomes
☐	☒	Estimates of effect sizes (e.g. Cohen's *d*, Pearson's *r*), indicating how they were calculated

Our web collection on statistics for biologists contains articles on many of the points above.

Software and code

Policy information about availability of computer code

Data collection	We used the Nikon NIS Elements software (version AR 5.21.03) to operate the Nikon Ti-E microscope that we used to collect all the data.
Data analysis	To design the FRET-FISH probes and analyze the FRET-FISH data described in this study we used custom MATLAB scripts available at https://doi.org/10.5281/zenodo.7081693. To automatically pick up FRET-FISH dots we used a custom package in MATLAB (DOTTER, v0.0.1) available at http://dx.doi.org/10.5281/zenodo.7112586. To analyze FRET-FISH signals on the active and inactive ChrX, we deconvolved the FRET-FISH images using our Deconwolf software available at https://github.com/elgw/deconwolf.

For manuscripts utilizing custom algorithms or software that are central to the research but not yet described in published literature, software must be made available to editors and reviewers. We strongly encourage code deposition in a community repository (e.g. GitHub). See the Nature Research guidelines for submitting code & software for further information.

Data

Policy information about availability of data

All manuscripts must include a data availability statement. This statement should provide the following information, where applicable:
- Accession codes, unique identifiers, or web links for publicly available datasets
- A list of figures that have associated raw data
- A description of any restrictions on data availability

The raw FRET-FISH images analyzed in this study have been deposited on Figshare under accession code 17080892 at https://doi.org/10.17044/scilifelab.17080892. A description of all the datasets is available in Supplementary Data 2. The MEFs ATAC-seq data used to validate FRET-FISH in this study are available in GEO under accession code GSE127926 at https://www.ncbi.nlm.nih.gov/geo/query/acc.cgi?acc=GSE127926. Source data for the main and supplementary figures are provided with this paper as a single .zip file.

Field-specific reporting

Please select the one below that is the best fit for your research. If you are not sure, read the appropriate sections before making your selection.

- [x] Life sciences
- [ ] Behavioural & social sciences
- [ ] Ecological, evolutionary & environmental sciences

For a reference copy of the document with all sections, see nature.com/documents/nr-reporting-summary-flat.pdf

Life sciences study design

All studies must disclose on these points even when the disclosure is negative.

Sample size	We did not perform any a priori sample size calculation. In each experiment, we aimed at imaging at least 1,000 cells, which we empirically found to be sufficient to obtain reproducible FRET-FISH score distributions.
Data exclusions	We excluded from downstream analyses FRET-FISH signal pairs (fluorescence dots in the donor and acceptor channel) detected in the same nucleus that were more than 7 pixels or 1.9 µm apart in 3D.
Replication	We performed all the experiments with chrX probes in three replicates and the experiments with chr18 probes in two replicates. All replicate experiments were successful.
Randomization	This study did not involve the treatment of human subjects or laboratory animals, therefore no randomization was applicable.
Blinding	We did not apply blinding since all the image analyses were performed in an unsupervised manner (automatic FISH dot picking).

Reporting for specific materials, systems and methods

We require information from authors about some types of materials, experimental systems and methods used in many studies. Here, indicate whether each material, system or method listed is relevant to your study. If you are not sure if a list item applies to your research, read the appropriate section before selecting a response.

Materials & experimental systems

n/a	Involved in the study
[ ]	[x] Antibodies
[ ]	[x] Eukaryotic cell lines
[x]	[ ] Palaeontology and archaeology
[x]	[ ] Animals and other organisms
[x]	[ ] Human research participants
[x]	[ ] Clinical data
[x]	[ ] Dual use research of concern

Methods

n/a	Involved in the study
[x]	[ ] ChIP-seq
[x]	[ ] Flow cytometry
[x]	[ ] MRI-based neuroimaging

Antibodies

Antibodies used	>>Anti-phospho-Histone H2A.X (Ser139, Merck Millipore, cat. no. 05-636)<br>>>Anti-53BP1 (Novus, cat. no. NB100-304)<br>>>Alexa Fluor 555 Donkey Anti-Mouse IgG (H+L) (Thermo Fisher Scientific, cat. no. A31570)<br>>>Alexa Fluor Plus 488 Donkey anti-Rabbit (Thermo Fisher Scientific, cat. no. A32790).
Validation	>>Anti-phospho-Histone H2A.X clone JBW301 (Ser139, Merck Millipore, cat. no. 05-636): validated for ChIP, ICC, IF, WB.<br>>>Anti-53BP1 (Novus, cat. no. NB100-304): validated for ChIP, Knockout-validated ICC, IF, IHC-Paraffin, IHC-Frozen, IP, Flow Cytometry, and Knockout-validated Western Blot.<br>>>Alexa Fluor 555 Donkey Anti-Mouse IgG (H+L) (Thermo Fisher Scientific, cat. no. A31570): validated for ICC/IF.<br>>>Alexa Fluor Plus 488 Donkey anti-Rabbit (Thermo Fisher Scientific, cat. no. A32790): validated for ICC/IF.

Eukaryotic cell lines

Policy information about cell lines

Cell line source(s)	We obtained mouse embryonic fibroblasts (MEFs) from ATCC (cat. no. SCRC-1040), NIH3T3 fibroblasts from ATCC (cat. no. CRL-1658) and HAP1 chronic myeloid leukemia cells from Horizon Discovery (cat. no. C859).

Authentication	None of the cell lines used in this study was authenticated.
Mycoplasma contamination	We periodically tested all the cell lines for mycoplasma contamination using the MycoAlert Mycoplasma Detection Kit (Lonza, cat. no. LT07-118). All cell lines tested negative for mycoplasma contamination.
Commonly misidentified lines (See ICLAC register)	None of the cell lines used in this study is registered in the International Cell Line Authentication Committee (ICLAC) database of misidentified cell lines.
